# Anti-MDA5 Antibody-Positive Dermatomyositis-Associated Interstitial Lung Disease With a False-Positive Tuberculosis-Targeted RNA Capture (TB-TRC) Result: A Multimodal Management

**DOI:** 10.7759/cureus.87004

**Published:** 2025-06-29

**Authors:** Miho Shibata, Toyoshi Yanagihara, Makoto Fujimoto, Naoko Himuro, Kenji Ito, Naoki Hamada, Takato Ikeda, Kosuke Masutani, Masaki Fujita

**Affiliations:** 1 Department of Respiratory Medicine, Fukuoka University Hospital, Fukuoka, JPN; 2 Department of Nephrology and Rheumatology, Fukuoka University Hospital, Fukuoka, JPN

**Keywords:** amyopathic dermatomyositis, anti-mda5 antibody, plasma exchange, tofacitinib, tuberculosis

## Abstract

Anti-MDA5 antibody-positive dermatomyositis often causes rapidly progressive interstitial lung disease (RP-ILD) with high mortality. We present a 59-year-old man with three weeks of fever and dyspnea whose chest CT images showed bilateral subpleural ground-glass and reticular opacities. A markedly elevated anti-MDA5 titer and skin biopsy confirmed anti-MDA5 anti-positive dermatomyositis-associated RP-ILD. Tacrolimus plus nintedanib was initiated. Initial bronchoalveolar lavage fluid (BALF) testing on admission was unexpectedly positive for *Mycobacterium tuberculosis* by targeted RNA capture (TB-TRC), precluding cyclophosphamide and prompting plasma exchange, followed by intravenous immunoglobulin (IVIG). Repeat BALF TB-TRC and all sputum cultures remained negative for *M. tuberculosis*, confirming a false-positive result. On day 28, tofacitinib replaced tacrolimus due to persistent hyperferritinemia. The patient was discharged without home oxygen therapy on day 54. This case highlights the importance of interpreting rapid assays in context and using multimodal therapy - steroids, calcineurin inhibition, antifibrotics, plasma exchange, IVIG, and Janus kinase (JAK) inhibition - for refractory anti-MDA5 RP-ILD.

## Introduction

Anti-MDA5 antibody-positive dermatomyositis frequently leads to rapidly progressive-interstitial lung diseases (RP-ILD), a condition with poor prognosis despite standard therapy [1]. Consequently, prompt exclusion of infectious etiologies is essential. Rapid molecular assays for pathogens, such as the tuberculosis-targeted RNA capture (TB-TRC) test, can detect mycobacterial RNA within hours, but they are sometimes prone to false positives when clinical suspicion is low [2,3]. Misinterpreting these results may delay life-saving immunosuppression or lead to unnecessary antimicrobial therapy, complicating treatment decisions. We describe a case of anti-MDA5 antibody-positive RP-ILD in which an initially false-positive TB-TRC result altered management, and we review how integrating assay characteristics with clinical judgment enabled successful, multimodal treatment.

## Case presentation

**Figure 1:**
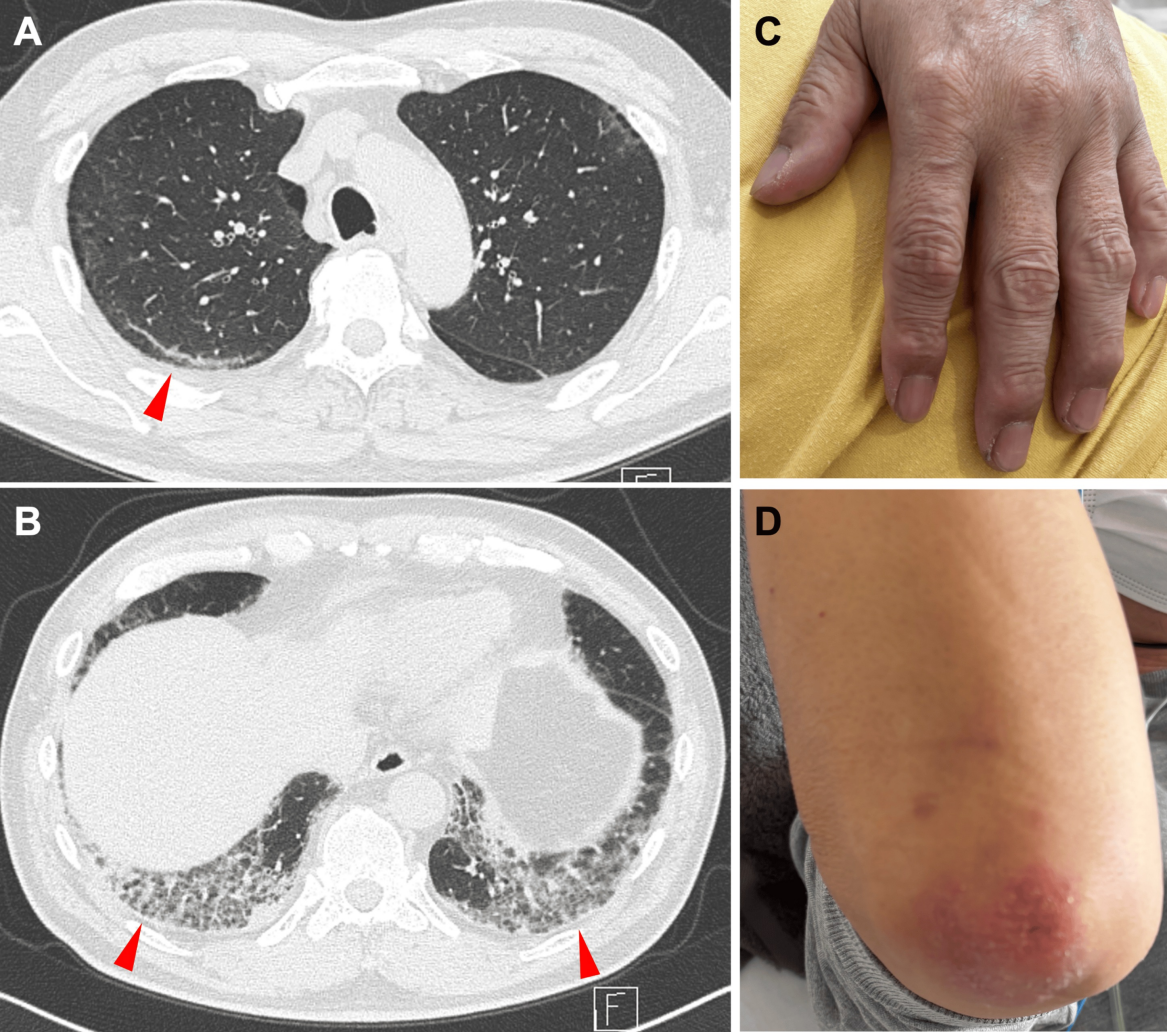
Findings at initial presentation (A) Axial high-resolution CT image at the level of the upper lobes demonstrates mild bilateral, patchy subpleural ground-glass opacities with subpleural curvilinear lines (arrowhead). (B) Axial high-resolution CT image at the level of the lung bases shows more extensive bilateral dorsal ground-glass and reticular opacities (arrowheads). (C) Clinical photograph of the patient’s left hand illustrating mild hyperkeratotic papules over the index finger and thumb. (D) Clinical photograph of the right elbow showing an erythematous, scaly papule over the extensor surface, representing a Gottron-like lesion.

A 59-year-old Japanese man was referred to our hospital for evaluation of progressive dyspnea and fever. His medical history was notable only for hypertension, treated with amlodipine. He had a 20-pack-year smoking history. There was no known history of autoimmune disease, ILD, or tuberculosis. He worked as a truck driver and had no obvious environmental or occupational exposures. Approximately three weeks before presentation, the patient developed low-grade fever (maximum 37.8°C), generalized fatigue, and a sore throat. He initially visited a local clinic and was prescribed oral antibiotics (garenoxacin) and antipyretics, but his symptoms did not improve. On the day before admission, he presented to a nearby hospital. A high-resolution computed tomography (HRCT) scan of the chest revealed bilateral, symmetric ground-glass opacities and consolidation predominantly in the dorsal and subpleural regions of all lobes, with a slightly patchy distribution (Figures 1A-1B). Subpleural curvilinear lines were also present. These findings were most consistent with an acute/subacute interstitial pneumonitis, raising suspicion for RP-ILD. Laboratory studies showed elevated inflammatory markers. For further evaluation and treatment, he was referred to our institution and admitted emergently.

On admission (hospital day 1), vital signs were as follows: temperature 37.6°C, blood pressure 128/76 mmHg, pulse 96 beats/min, respiratory rate 17 breaths/min, and oxygen saturation 94% on 1 L/min supplemental oxygen via nasal cannula. Auscultation of the lungs revealed fine crackles bilaterally, most prominent at the lung bases. Cardiovascular, abdominal, and neurologic examinations were unremarkable. There was no peripheral lymphadenopathy. Discrete erythematous, hyperkeratotic papules were noted over the index finger and thumb on both hands (Figure 1C). Subungual hemorrhages were present on the middle finger of the right hand. Mild, patchy desquamation was observed along the nasolabial folds. Erythematous, scaly papule over the extensor surface was also noted, representing a Gottron-like lesion (Figure 1D). There was no heliotrope rash or shawl sign. There was no objective muscle weakness on manual muscle testing (Medical Research Council grade 5/5 in all proximal and distal muscle groups). No myalgia or arthralgia was reported.

**Figure 2:**
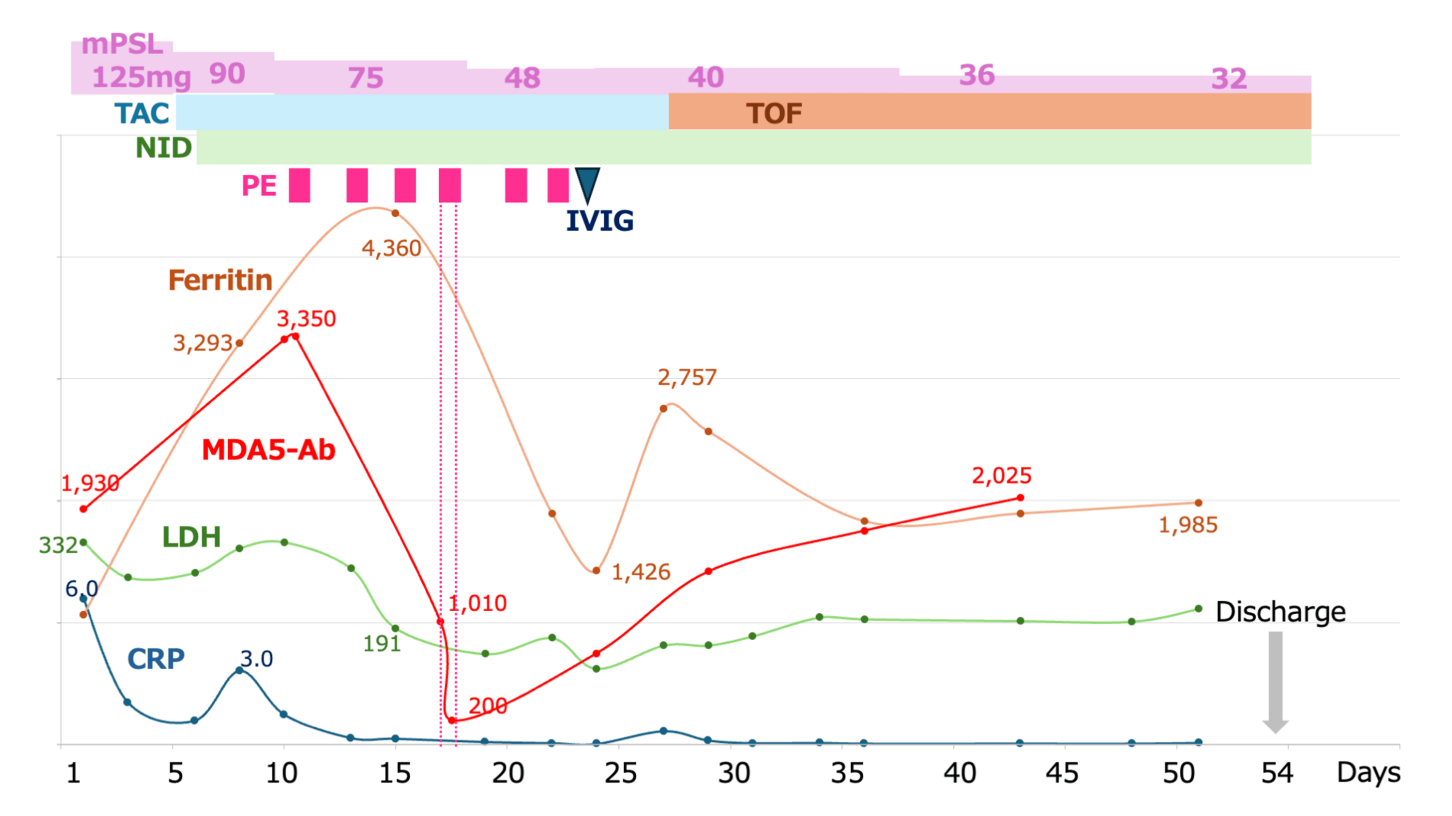
Clinical course of the patient Days from admission (day 1) to day 54 are shown on the x-axis. Top bars indicate therapies: methylprednisolone (mPSL); tacrolimus (TAC); tofacitinib (TOF); nintedanib (NID); plasma exchange sessions (PE); intravenous immunoglobulin (IVIG). Line plots show C-reactive protein (CRP; navy, left y-axis), LDH (green, left y-axis), ferritin (orange, right y-axis), and anti-MDA5 antibody titer (red, right y-axis). The patient was discharged on day 54.

On hospital day 1, the following laboratory values were obtained: C-reactive protein (CRP) 5.95 mg/dL (reference: <0.3 mg/dL), ferritin: 1,067 ng/mL (reference: 20-300 ng/mL), Krebs von den Lungen-6 (KL-6): 798 U/mL (reference: <500 U/mL). Fiberoptic bronchoscopy was performed. Bronchoalveolar lavage fluid (BALF) was collected from the right lower lobe bronchus (right B8). BALF analysis revealed 90% macrophages, 6% lymphocytes, 1% neutrophils, and 3% ciliated cells. The CD4/CD8 ratio was 0.4. FilmArray Respiratory Panel 2.1 (BioFire Diagnostics, Salt Lake City, UT, USA) revealed negative for influenza, adenovirus, SARS-CoV-2, and Mycoplasma. Unexpectedly, TB-TRC was positive, although the smear was negative. Bacterial cultures revealed normal flora. Cytology revealed no malignant cells. Because of the positive TB-TRC result, the patient was placed under airborne isolation pending confirmation. A punch biopsy of an erythematous papule on the dorsal index finger was also performed. The patient was initiated on 125 mg of methylprednisolone (mPSL) along with minocycline for potential ILD and bacterial infection (Figure 2).

On day 5, his oxygenation worsened compared to admission, and he required 2 L/min of supplemental oxygen via nasal cannula at rest. The anti-MDA5 antibody assay submitted on admission returned with an exceedingly high titer (1,930 index). Antinuclear antibody testing was negative at a titer of <1:40, and anti-aminoacyl-tRNA synthetase (anti-ARS) antibodies were negative. The T-SPOT.TB assay (Oxford Immunotec, Abingdon, UK) returned negative. Skin biopsy findings were consistent with dermatomyositis. He was diagnosed with anti-MDA5 antibody-positive dermatomyositis-associated RP-ILD. Tacrolimus, targeting a trough concentration of 10-15 ng/mL, and nintedanib were initiated. Because bacterial pneumonia was deemed unlikely to contribute significantly, minocycline was discontinued.

On day 8, his oxygen requirement remained at 2 L/min, but CRP, lactate dehydrogenase (LDH), and ferritin had risen; ferritin reached 3,293 ng/mL, indicating refractory disease. Given the positive TB-TRC result, intravenous cyclophosphamide (IVCY) was avoided, and plasma exchange was chosen instead. Plasma exchange was started on day 10. Following this intervention, CRP, LDH, and ferritin peaked on day 15 and subsequently declined, and oxygenation began to improve. Plasma exchange was performed three times per week for a total of six sessions and was discontinued on day 22. In each session, approximately 4.5 L of plasma (one plasma volume) was exchanged and replaced with 4 L of 5% albumin solution and 0.5 L of fresh-frozen plasma.

On day 16, repeat bronchoscopy with targeted TB-TRC testing at the previously positive site was negative. All three sputum and gastric aspirate TB-TRC tests obtained during hospitalization were negative. The initial TB-TRC positivity was therefore considered a false positive, and airborne isolation was lifted. Subsequent cultures for *Mycobacterium tuberculosis* remained negative throughout the clinical course.

**Figure 3:**
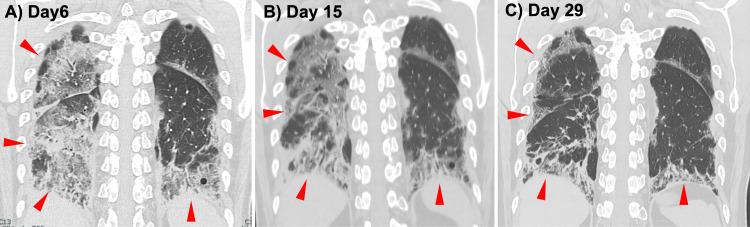
Serial coronal chest CT images over the clinical course (A) Day 6: Extensive bilateral subpleural ground-glass opacities and consolidation, most pronounced in the right lower lobe (arrowheads). (B) Day 15: Decreased ground-glass attenuation with persistent reticulation and early traction bronchiectasis (arrowheads). (C) Day 29: Near resolution of ground-glass changes with established fibrotic reticulation and volume loss in the right lower lobe (arrowheads).

On day 23, 40 g of intravenous immunoglobulin (IVIG) was administered for hypogammaglobulinemia after plasma exchange. By day 28, although his oxygenation remained stable, ferritin had again increased to 2,757 ng/mL, indicating treatment resistance. Tacrolimus was switched to tofacitinib 10 mg daily, after which ferritin peaked once more and then declined. Throughout this period, corticosteroids were tapered gradually. His serum ferritin had plateaued, but his oxygenation was maintained on room air, and imaging showed no worsening (Figure 3). Given his stable respiratory status and prolonged hospitalization, he was discharged home on day 54 and continues outpatient follow-up and treatment. At discharge and throughout outpatient follow‑up, there were no clinical or radiographic signs suggestive of active tuberculosis.

## Discussion

This case of anti-MDA5 antibody-positive RP-ILD is notable for the diagnostic and therapeutic challenges posed by an initial false-positive tuberculosis test. The primary lesson from this case is the critical importance of interpreting rapid molecular assays within the patient's broader clinical context. Although TB-TRC has a reported sensitivity of 90% and specificity of 98% [3], its positive predictive value decreases significantly when clinical suspicion is low. False-positive results can arise from laboratory contamination, or cross-reactivity with nontuberculous mycobacteria, or occasional borderline or nonspecific amplification inherent to the assay design [4,5]. This result necessitated a deviation from standard aggressive immunosuppression, specifically deferring cyclophosphamide, and instead utilizing plasma exchange as a bridging therapy while a definitive infectious workup was completed. This experience highlights a pragmatic approach for managing critically ill patients with autoimmune emergencies when a concurrent infection is suspected but not confirmed.

The epidemiology and natural history of anti-MDA5 RP-ILD justify an early, aggressive, multimodal immunosuppressive approach. Anti-MDA5 antibodies are detected in 30-50% of dermatomyositis-associated ILD cases, often presenting as clinically amyopathic dermatomyositis with minimal muscle involvement but rapidly progressive lung injury [6-8]. Standard therapy combining high-dose corticosteroids with calcineurin inhibitors, cyclophosphamide, Janus kinase (JAK) inhibitors, or IVIG is necessary, yet approximately 30% of patients remain treatment-resistant and die within six months [8]. Our patient presented with multiple established risk factors for a poor prognosis, including highly elevated serum ferritin, hypoxemia, mandating an immediate and robust therapeutic response [7-10].

Our therapeutic strategy evolved in response to both the diagnostic uncertainty and the patient's clinical trajectory. Plasma exchange served a critical dual purpose: it offered a potent, non-cytotoxic intervention while the risk of tuberculosis was being clarified, and it provided salvage therapy for refractory inflammation. By removing pathogenic autoantibodies, cytokines, and immune complexes [11,12], the six sessions of plasma exchange led to a marked improvement in oxygenation and inflammatory markers, supporting its use as a first-line tool to rapidly control hyperinflammation when cytotoxic agents are contraindicated [13-16]. Concurrently, the early introduction of the antifibrotic agent nintedanib on day 6 reflects a proactive strategy to mitigate irreversible fibrotic progression without compounding the immunosuppressive burden [17,18].

The subsequent switch from a calcineurin inhibitor (tacrolimus) to a JAK inhibitor (tofacitinib) represents a strategic pivot in the immunosuppressive regimen. This change was prompted by rising ferritin and anti-MDA5 titers on day 27, suggesting that the initial regimen was insufficient. The American College of Rheumatology (ACR) and the American College of Chest Physicians (CHEST) guidelines conditionally recommend JAK inhibition or cyclophosphamide in progressive cases [19]. Tofacitinib was selected for its mechanism of targeting the type I interferon signaling pathway, a key driver in dermatomyositis pathogenesis. Tofacitinib use is supported by multicenter, retrospective data suggesting superior survival outcomes compared to calcineurin inhibitors [20,21]. This decision was also weighed against the use of cyclophosphamide. While cyclophosphamide is a conditionally recommended option [19,22], we ultimately avoided it due to the initial concern for infection and the patient's age, which places him at a higher long-term risk for secondary malignancy. Further, larger real-world data have not definitively confirmed the benefit of high-dose IVCY combined with steroids and calcineurin inhibitors [23]. Switching to tofacitinib rather than adding it to tacrolimus was a deliberate choice to avoid excessive T-cell suppression and the associated risk of opportunistic infections, such as cytomegalovirus.

## Conclusions

This case illustrates a stepwise, adaptive treatment algorithm for anti-MDA5 RP-ILD: begin with high-dose steroids and calcineurin inhibition; consider early antifibrotic therapy; reserve plasma exchange in refractory, special-circumstance scenarios; and employ JAK inhibitors for potential relapse. Recognition of test limitations, such as false-positive TB-TRC results, is essential to avoid misdirected therapy. Further prospective studies are needed to refine optimal combinations, sequences, and timing of immunosuppressive and antifibrotic modalities in this high-mortality disease.
